# Water sources and mixing in riparian wetlands revealed by tracers and geospatial analysis

**DOI:** 10.1002/2015WR017519

**Published:** 2016-01-28

**Authors:** Jason S. Lessels, Doerthe Tetzlaff, Christian Birkel, Jonathan Dick, Chris Soulsby

**Affiliations:** ^1^ Northern Rivers Institute, School of Geosciences, University of AberdeenAberdeenUK

**Keywords:** tracer, geostatistics, synoptic sampling, biogeochemistry

## Abstract

Mixing of waters within riparian zones has been identified as an important influence on runoff generation and water quality. Improved understanding of the controls on the spatial and temporal variability of water sources and how they mix in riparian zones is therefore of both fundamental and applied interest. In this study, we have combined topographic indices derived from a high‐resolution Digital Elevation Model (DEM) with repeated spatially high‐resolution synoptic sampling of multiple tracers to investigate such dynamics of source water mixing. We use geostatistics to estimate concentrations of three different tracers (deuterium, alkalinity, and dissolved organic carbon) across an extended riparian zone in a headwater catchment in NE Scotland, to identify spatial and temporal influences on mixing of source waters. The various biogeochemical tracers and stable isotopes helped constrain the sources of runoff and their temporal dynamics. Results show that spatial variability in all three tracers was evident in all sampling campaigns, but more pronounced in warmer dryer periods. The extent of mixing areas within the riparian area reflected strong hydroclimatic controls and showed large degrees of expansion and contraction that was not strongly related to topographic indices. The integrated approach of using multiple tracers, geospatial statistics, and topographic analysis allowed us to classify three main riparian source areas and mixing zones. This study underlines the importance of the riparian zones for mixing soil water and groundwater and introduces a novel approach how this mixing can be quantified and the effect on the downstream chemistry be assessed.

## Key Points

- Estimate the extent and dynamics of source waters in a riparian area
- Use geospatial information to estimate the spatial extent of source waters
- Source waters are highly dynamic and strongly influence by climatic conditions

## 1 Introduction

It has long been recognized that knowledge of spatial patterns and temporal variations in tracer dynamics improves our understanding of the hydrological response of catchments [e.g., *Hewlett and Hibbert*, 1967]. Different tracers provide different complementary information in this regard: various biogeochemical tracers and stable isotopes can help constrain the sources of runoff and their temporal dynamics [*Kirchner et al*., 2010; *Lischeid*, 2008; *Neal et al*., 1997]. For example, stable isotopes such as deuterium (δ^2^H) are commonly used to indicate sources of water, with depleted values indicating groundwater sources and enriched δ^2^H indicating biological or evaporative fractionation [*Kendall and McDonnell*, 2012; *Kirchner et al*., 2010; *Soulsby et al*., 2007]. Likewise, geochemical tracers such as alkalinity can correlate with subsurface contact time, indicative of groundwater sources while dissolved organic carbon (DOC) concentrations reflect sources in biologically active, organic‐rich upper soil horizons [*Boyer and Hornberger*, 1997; *Inamdar and Mitchell*, 2006]. The development of laser spectroscopy techniques has recently made the analysis of stable water isotopes much more rapid and inexpensive [*Koehler and Wassenaar*, 2011; *Penna et al*., 2010]. Many studies have utilized this to increase the temporal resolution of sampling to improve insights into hydrological function [*Birkel et al*., 2014; *Kirchner and Neal*, 2013; *Kirchner et al*., 2000]. *Zimmer et al*. [2013] used high‐resolution synoptic surveys of stream chemistry to highlight the importance of soil distribution and the location of groundwater seepages on downstream hydrochemical changes in a small (41 ha) catchment. Additionally, *Peralta‐Tapia et al*. [2015] revealed the importance of the spatial distribution of differing source waters on stream water using a synoptic sampling in the Krycklan Catchment in northern Sweden. The results of these studies provide valuable insights in the interaction between surface and stream water. However, the use of repeated synoptic surveys of surface waters under variable antecedent conditions offers the ability to assess the temporal dynamics of these processes.

To date, inferences of changes in downstream water chemistry [*Likens and Buso*, 2006; *Soulsby et al*., 2004; *Temnerud and Bishop*, 2005] have been mostly provided, with studies highlighting the variability in source areas and how this variability affects downstream chemistry [*Ågren et al*., 2014; *Bishop et al*., 2008; *Grabs et al*., 2012; *Zimmer et al*., 2013]. These findings have led to the development of dynamic models to represent these processes [*Barling et al*., 1994; *Beven and Freer*, 2001; *Birkel et al*., 2010]. The importance of structural landscape units such as riparian zones [*Jencso et al*., 2009; *McGlynn et al*., 2004; *Seibert et al*., 2009; *Smart et al*., 2001; *Tetzlaff et al*., 2014] and wetlands [*Bishop et al*., 2008; *Lidman et al*., 2014] in controlling downstream chemistry has been highlighted. However, the direct monitoring of sources of water is usually limited to relatively few sampling points and the high‐resolution spatial distribution of processes is usually poorly characterized [*Grabs et al*., 2012; *Orlowski et al*., 2015; *Seibert et al*., 2009; *Tetzlaff et al*., 2014].

Geostatistical methods provide the potential to better assess spatially distributed observations and extrapolate properties through space based on the spatial structure of point observations [*Bivand et al*., 2008]. The general approach of these methods has been to use line of sight (Euclidean) distances between observations, which are inappropriate for within stream networks. Recent developments in the application of spatial statistics to river networks to assess spatial organization of catchments [*Hoef et al*., 2006; *Peterson and Hoef*, 2010] facilitate the assessment of the importance of ecosystem functioning at different spatial scales based on stream structures. This ability to examine the spatial structure along stream networks provides the opportunity to address the research gap in capturing spatial and temporal variability in runoff generation sources [*Hoef et al*., 2006]. More recently, *McGuire et al*. [2014] used these methods to examine tracers throughout a stream network to identify hydrological active areas within the network. Geostatistical methods, used in conjunction with spatially high‐resolution environmental tracer applications, thus provide a unique opportunity to assess heterogeneity in riparian zones and identify areas which are associated with groundwater seepages, soil water drainage, and areas where mixing of these waters is common. This provides a basis to extrapolate and assess hydrological processes at the landscape and catchment scale.

The Burntland Burn long‐term experimental catchment has been the focus of numerous tracer‐aided studies which have revealed the dominant influence of an extensive riparian peatland on the hydrology of the catchment [*Birkel et al*., 2011a, 2011b; *Dick et al*., 2015; *Geris et al*., 2014]. This riparian zone has been shown to have a large influence on the mixing of soil water and groundwater on downstream stream chemistry [*Tetzlaff et al*., 2014; *Birkel et al*., 2015]. Here we apply geostatistical techniques to a high spatial resolution, repeat sampled synoptic data set of multiple tracers measured across surface waters of this extended riparian wetland. Utilizing high‐resolution LiDAR‐derived landscape characteristics and geostatistical techniques, we explore the spatial distribution of runoff generation sources based on tracer signatures to understand how catchment dynamics and heterogeneity affect flow paths and runoff generation within the riparian zone. Therefore, the specific research questions of this study are:

1. What can different tracers reveal about the spatial heterogeneity of water sources within an extended riparian zone?
2. Can geostatistical tracer analysis, in combination with high‐resolution LiDAR‐derived spatial properties, be used to classify source areas and mixing zones within the riparian zone?
3. How do variable hydroclimatic conditions affect the spatial patterns of water sources and the hydrological functioning at the catchment scale?

## 2 Data and Methods

### 2.1 Study Location and Sampling Description

**Figure 1:**
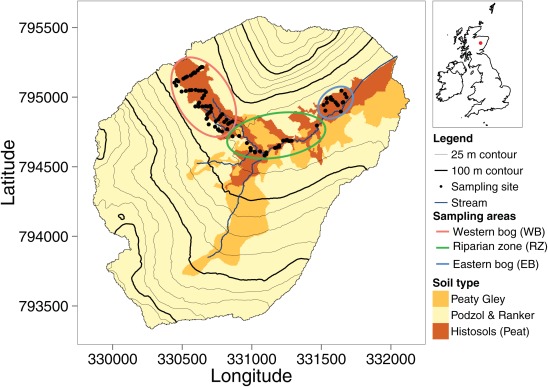
Location and dominant soil classes of the study catchment, showing locations of the sampling sites.

**Figure 2:**
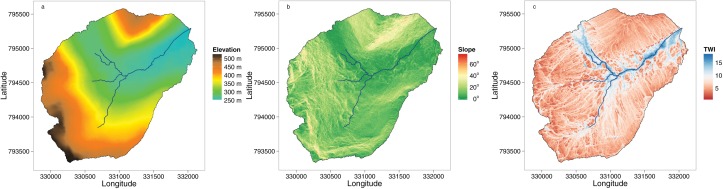
LiDAR‐derived spatial products; (a) elevation, (b) slope, and (c) SWI showing the extent of the permanently saturated riparian wetland in blue.

The 3.2 km^2^ Burntland Burn study catchment (Figure 1) is located in north‐east Scotland. A detailed description of the catchment can be found elsewhere [e.g., *Birkel et al*., 2011a, 2010; *Soulsby et al*., 2007] . The catchment is characterized by a temperate/boreal, humid climate with a mean air temperature of 7.4°C [*Hannah et al*., 2008]. Annual rainfall of ∼1000 mm is distributed evenly throughout the year with an annual mean stream discharge of 1.8 mm d^−1^. The glacial history of the catchment has a large influence on the topography (Figures 2a and 2b) and related distribution of soils, with deep organic peaty soils occurring in the wide valley bottoms and with freely draining podzols dominating on the hillslopes (Figure 1). Around 70% of the catchment is covered by glacial drift deposits. Recent geophysical surveys have shown that these are typically ∼5m deep on the steeper hillslopes and up to 40m deep in the valley bottoms (*Soulsby et al.,* 2015) . These cover bedrock geology of granite in the north and east, with schists in the west. In places, the schists contain meta‐limestones.

The wetlands in the valley bottom are commonly saturated with surface ponding of water. However, the extent of this saturated area is dynamic and heavily dependent on antecedent conditions; observations have shown that it can range from 3 to 40% of the catchment [*Birkel et al*., 2010]. Sampling of surface waters in the saturated area was undertaken on three occasions at 94 sites (Figure 1) with an additional 10 sampling locations chosen along the stream network. Sampling of the pooled water was undertaken when possible; sampling of some specific locations was not possible on some drier occasions due to a lack of water. Three main zones were sampled within the catchment valley bottom (Figure 1): two peat bog areas were sampled, (i) the western bog (WB) area located in the north‐west of the catchment and (ii) the eastern bog (EB) located north of the main stream and close to the catchment outlet. The third area focused on the connected pools of water within the riparian zone (RZ) between the WB and the EB areas.

For each of the campaigns, all samples were analyzed for deuterium (δ^2^H), gran alkalinity (GA), and dissolved organic carbon (DOC). Measurements of δ^2^H ratios were determined using a Los Gatos Research (LGR, California, USA) DLT‐100 laser diode water isotope analyzer according to the Vienna Standard Mean Ocean Water (VSMOW) standards. Instrument precision for δ^2^H was ±0.4‰. GA was measured following *Neal et al*. [1997] using acidimetric titration (using 0.005 *M* H_2_SO_4_). DOC was determined using a PPM LABTOC Analyzer after 0.45 µm filtration of each sample was undertaken.

In May 2013, an Airborne Research and Survey Facility (ARSF) coordinated survey was undertaken to obtain a detailed digital elevation model (DEM) of the catchment. The survey collected Light Detection and Ranging (LiDAR) data that were processed using LASTOOLS [*Isenburg and Schewchuck*, 2007] and GRASS GIS [*GRASS DevelopmenTeam*, 2015] to derive a 1 m^2^ DEM. Based on the DEM, several hydrologically based topographic indices were derived using a combination of the GRASS GIS and SAGA [*SAGA*, 2014] software products. The indices included: elevation, slope, aspect, contributing area (CA), SAGA wetness index (SWI) [*Böhner and Selige*, 2006], topographical roughness index (TRI) [*Riley et al*., 1999], and topographical position index (TPI) [*Guisan et al*., 1999]. Figure 2 shows the detail of the LiDAR‐derived DEM, slope, and the SWI, with the valley bottoms of the catchment characterized with a low slope and high SWI.

### 2.2 Statistical Analysis

To gain an increased understanding of the spatial heterogeneity and patterns of the tracers throughout the extended riparian wetland zone, several geospatial analyses were undertaken. The statistical analyses examined the potential relationship between the topographical indices derived from the DEM and the sampled tracer observations to provide spatial estimates of concentrations. Using the spatial estimates, a *k*‐means clustering approach was undertaken to classify areas of water sources based on the three tracers for each sampling campaign.

The methods used to make spatial estimates of each tracer are similar to those of *Saby et al*. [2011] and are summarized as

1. Transform the observations of each tracer to ensure that the data are normally distributed.
2. Fit a linear model to each tracer for each sampling campaign using step‐wise backward elimination.
3. Model the variogram of the linear model residuals using a Dowd estimator and fit a Matérn function to this using a weighted least squares [*Cressie*, 1985].
4. Spatially predict each tracer by combining the linear estimates with kriged estimates of the predicted residuals of the linear model.
5. Back transform each of the predictions to the original scale.

#### 2.2.1 Spatial Model Development

For each of the sampled tracers on each sampling campaign, the ability of the topographic indices to account for variation of the tracers was examined. The structure of this model can be written as;

(1) z=Xβ+η

where z is one of the three tracers, X is a *n × p* design matrix containing the topographical indices (fixed effect), and β is a vector of size *p*. As the tracer samples represent a random spatial trend, it was assumed that the residuals (random effect) of the model η∼N(0,V) are spatially correlated with a Gaussian distribution and a covariance matrix V [*Rawlinsand Marchant*, 2009]. In this study, the observed tracers were positively skewed and were transformed prior to the fitting of the linear model. Both δ^2^H and GA were transformed using a square root transformation, and DOC was transformed using a log transformation. Linear models were fitted using step‐wise backward elimination based on the full model which included all topographical indices, where the final model was selected based on the lowest Akaike Information Criterion (AIC) [*Akaike*, 1973]. This provides an assessment of the goodness of fit in relation to the number of predictor variables included in the model. In instances where no topographic indices remained in the final model, the mean of the transformed tracer was used for estimation.

The random effect of each model was assumed to be spatially correlated and therefore, this correlation was estimated using a variogram model. However, as one of the aims of the spatial sampling was to identify zones of importance in relation to geographic sources of water, there was a high likelihood of potential outliers caused by hydrologically relevant landscape structures (e.g., groundwater seeps) within the observed tracers. The presence of outliers can have a detrimental influence on the use of estimation of the variogram [*Lark*, 2002] and therefore robust methods must be used to account for this potential effect. To account for this, the Dowd estimator [*Dowd*, 1984] was used to represent the variogram of the random effect as it has been shown to be a robust method in the presence of very large or small values within the observations [*Lark*, 2010]. The variogram of the random effect was modeled using a Matérn variogram model [*Matérn*, 1960], where the spatial semivariance ( $\gamma \left(h\right)$) between two observations is represented by the following function

(2) $$\gamma \left(h\right)\;=\;c_{o}+c_{1}\left\{1-\frac{1}{2^{V-1}}\Gamma \left(v\right){\left(\frac{h}{\phi }\right)}^{v}K_{v}\left(\frac{h}{\phi }\right)\right\}\;for\;h>0,$$

$$\gamma \left(h\right)\;=\;0\;for\;h=0$$

where *h* is the Euclidean distance between two sampling locations, *c*_0_ is the nugget of the model (unexplained spatial variance), *c*_1_ represents the spatial variance of the model, (*c*_0_ + *c*_1_) is equivalent to the overall variance of the model, ϕ is the range parameter of the model, and $K_{v}$ is a Bessel function of the second kind of order v and a gamma function, Γ [*Rawlins and Marchant*, 2009]. The variogram was fitted using Dowd's estimator using the georob package [*Papritz et al*., 2014] and the Matérn function was estimated using the gstat package [*Pebesma*, 2004] in the R programming language [*R Core Team*, 2012].

#### 2.2.2 Spatial Estimates and Outlier Detection of Tracers

Spatial estimates of each tracer were based on a combination of the approximations made using the fixed effects from the linear model and kriged estimates of the random effect. As the spatial dynamics of the saturated zone result in variability in the extent and longevity of pooled water in the valley bottom it was not possible to predict the locations of these areas. Therefore, given the near‐permanent saturation in the riparian area, the spatial estimates of each tracer represent the expected value if pooled water was present at that point in time. Fixed effect estimates were made using the linear model fitted using step‐wise backward elimination and were combined with the kriged estimates of the random effect. The spatial estimates were made using the gstat package and back‐transformations were undertaken using the geoR package [*Diggle and Ribeiro*, 2007] within the R programming language.

Estimates of the random effect from the leave‐one‐out‐cross‐validation (LOOCV) method allow for the investigation of observations which differ from the surrounding observations, based on the spatial variogram model. *Lark* [2002] explains how the standardized error (SE) of the LOOCV process can be used to detect spatial outliers. A threshold must be selected to determine if observations are deemed to be spatial outliers. In this study, observations were classified as spatial outliers if the SE was greater than 1.96 or less than −1.96.

#### 2.2.3 Model Evaluation and Validation

The accuracy of the model predictions for each tracer was assessed with the root‐mean‐square error (RMSE), *r* ^2^, and the percent bias (PBIAS). The accuracy of the random effect was estimated using the LOOCV analysis and these estimates were added to those of the linear model. An assumption of the kriging predictions is that the predictions from this method will have a Gaussian distribution [*Marchant et al*., 2010]. This assumption can be assessed using LOOCV, as the standardized squared prediction errors (SSPE) from this method should be *X* ^2^ with 1° of freedom, a mean close to 1.0, and a median of 0.455 [*Lark*, 2002].

### 2.3 Classification of Water Sources and Mixing

To simplify the interpretation and examine the temporal dynamics and spatial distribution of the water in the valley bottom of the catchment, *k*‐means classification was undertaken using the tracer estimates. The spatial estimates of each tracer were standardized and classified into three classes of water sources using the *k*‐means clustering method separately for each sampling campaign. Previous work within the catchment has shown that groundwater (depleted deuterium, high alkalinity, and low DOC), soil water (enriched deuterium, low alkalinity, and high DOC), and a mixture of these two types or classes of water are the main types of water within the catchment.

## 3 Results

The repeated synoptic sampling reveals valuable information about the spatial and temporal trends within the larger riparian zone of the catchment. The results reveal the influence the climatic conditions have on the spatial variability through the extended riparian zone. The geostatistical analysis revealed no consistent trend with the included LiDAR‐derived covariates.

### 3.1 Hydroclimatic Context During Sampling Campaigns

**Figure 3:**
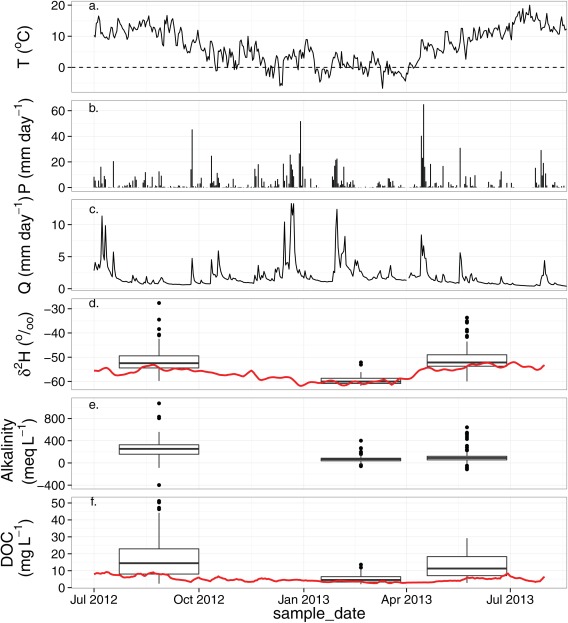
Hydroclimatic data and summary of tracer data during the study period. (a) Air temperature, (b) precipitation, and (c) stream discharge; Tukey box plots for spatial data on each sampling date. Whiskers represent 1.5 * interquartile range as specific in the ggplot2 package [*Hadley*, 2009]. (d) Deuterium, (e) gran alkalinity, and (f) DOC; red lines indicate weekly mean for each tracer at outlet.

Figure 3 provides an overview of the temporal hydroclimatic conditions and the variations of each of the tracers during the study period. Table 1 provides a summary of the saturated area samples collected during the three sampling campaigns. The three sampling campaigns were undertaken at times with marked antecedent hydroclimatic differences on each occasion. The first sampling campaign was undertaken during late summer (August 2012) when the catchment was relatively dry after several weeks of little precipitation and very small discharge responses. This spatial survey revealed enriched δ^2^H, high alkalinity values, and high DOC concentrations with mean values of −51.6‰, 258.2 µeq L^−1^, and 18 mg L^−1^, respectively, and the largest variation of the three sampling campaigns. The winter survey (February 2013) was undertaken during wet and cool conditions characterized by depleted δ^2^H, low alkalinity, and low DOC concentrations with mean values of −59.7‰, 73 µeq L^−1^, and 5.1 mg L^−1^, respectively. This sampling survey corresponded with the smallest variation of the three sampling surveys with standard deviations of 1.7‰, 63 µeq L^−1^, and 2.3 mg L^−1^ for δ^2^H, alkalinity, and DOC. The final sampling survey occurred during early summer (May 2013), when temperatures were mild and the catchment was relatively wet. During this survey, the tracers had relatively small variability with standard deviations of 5.1‰, 122 µeq L^−1^, and 7 mg L^−1^ for δ^2^H, GA, and DOC, respectively.

**Table 1:** Summary Statistics of the Saturation Area Samples for Each Sampling Campaign

Variable	Sample Date	Minimum	Maximum	Mean	Standard Deviation	*n*
Deuterium (^o^/_oo_)	Aug 2012	−59.8	−27.6	−51.6	5.4	83
	Feb 2013	−61.8	−52.2	−59.7	1.7	92
	May 2013	−60	−33.7	−50.9	5.1	94
Gran alkalinity (µeq L^−1^)	Aug 2012	−398.3	1072.1	258.2	183	83
	Feb 2013	−58.7	401.7	73.1	63.2	94
	May 2013	−114.7	641	110.6	122.1	93
DOC (mg L^−1^)	Aug 2012	2.4	51.1	18	12.3	86
	Feb 2013	2	13.6	5.1	2.3	94
	May 2013	2.8	29.2	12.9	7	60

Observations of δ^2^H and DOC and estimates of GA at the outlet provided additional information about the mixture of the different source waters leaving the catchment (shown as weekly mean values in Figure 3). There was a clear seasonal trend for δ^2^H, with summer enrichment and winter depletion. The observed values at the outlet reflect a similar trend to that of the distribution of the spatial surveys. Estimated GA at the catchment outlet tended to have a higher concentration compared with the median of the spatial surveys. Additionally, there was a general trend of GA increasing during the dry warm 2012 summer. The trend of DOC at the catchment outlet also indicated a seasonal pattern with higher concentrations seen during warmer months. This trend of DOC at the catchment outlet was similar to that of the spatial surveyed points, but the concentration at the outlet was less than that of the spatially sampled mean DOC. However, recent work has revealed that observed DOC loads in the catchment show less seasonality as the seasonality is present in stream discharge [*Dick et al*., 2015].

### 3.2 Spatial Heterogeneity of Multiple Tracers and Controlling Landscape Structures

**Figure 4:**
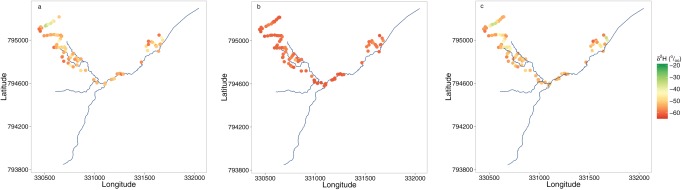
Observed deuterium values of each sampling campaign; (a) August 2012, (b) February 2013, and (c) May 2013.

Figure 4 shows the spatial distribution of δ^2^H during the three sampling campaigns and provides an overview of the temporal variation between the sampling surveys. During the late summer sampling campaign (August 2012), strong spatial differentiation and disconnection could be observed, with enriched deuterium values observed in pools on the WB and in the RZ. Contrasting this, several depleted deuterium values were found in areas which are known for groundwater seepages at the interface of the RZ and the surrounding hill slopes. Much of this spatial differentiation was not present during the winter sampling campaign, when the catchment was under wet conditions and the sources and different waters were well connected with a similar composition. Samples collected in the early summer sampling campaign revealed that the spatial differentiation had reestablished with δ^2^H values being more enriched than during the winter. By then, the well‐connected source waters appeared to be starting to become as differentiated as the previous summer sampling campaign as drying increased in the warmer summer.

Based on step‐wise backward elimination, the fixed effect coefficients of the final model for each of the tracers are summarized in Table 2. The use of backward elimination facilitates the identification of the main controlling topographical indices of each of the spatial patterns for the three tracers. It also presents the fitted values of the Matérn function of the spatial‐dependent random effects. The results show that TPI and the CA topographical indices were dropped from all of the models, indicating that these did not have a significant influence on the observed tracers. The topographical wetness index (SWI) only remained in the one of the fitted models. It was found to have a significant effect on δ^2^H during winter (February 2013), indicating the influences of SWI on δ^2^H during wetter periods, when there was little spatial variation of the tracers. Elevation was the only fixed effect determined to be a significant topographical index for DOC and was also included in all GA models.

**Table 2:** Summary of the Fixed Effect Coefficients ( β) for the Model Intercept ( $\beta_{0}$), Each Topographical Index and Parameters of the Random Effects Matérn Function

Variable	Sample Date	$\beta_{0}$	$\beta_{slope}$	$\beta_{TRI}$	$\beta_{SWI}$	$\beta_{ELEV}$	$c_{0}$	$c_{1}$	Effective Range (m)
Deuterium (^o^/_oo_)	Aug 2012	4.77^a^		−19.57^a^			0	3.05	53.91
	Feb 2013	8.55^a^	−0.19^a^		−0.19^a^	−0.02	0.52	0.48	223.14
	May 2013	4.53^a^	−0.21^a^				0.36	2.23	61.63
Gran alkalinity (µeq L^−1^)	Aug 2012	98.84^a^				−0.19	8.87	28.92	64.15
	Feb 2013	50.98^a^		22.92		−0.12	8.76	12.67	91.83
	May 2013	64.69^a^				−0.14	13.88	18.09	73.53
DOC (mg L^−1^)	Aug 2012	−5.48^a^	−0.09^a^			0.03^a^	0.19	0.19	81.99
	Feb 2013	−0.89		−3.36^a^		0.01^a^	0.04	0.19	151.17
	May 2013	2.59^a^		−2.71			0.08	0.3	71.59

a indicates 0.95 significance of fixed effects.

**Figure 5:**
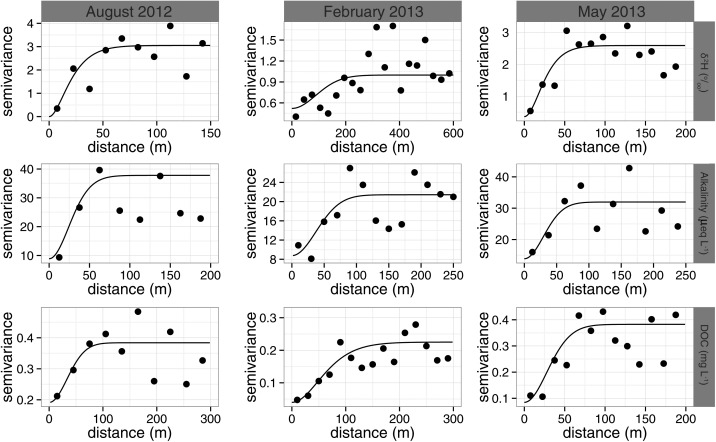
Fitted variogram models of the random error of each variable on the three sampling campaigns. Columns indicate the sampling campaign with rows indicating the variable.

Figure 5 shows the variogram models of the random effect for each of the tracers with the parameters of each of the variogram models summarized in Table 2. Visually, the Matérn function appears to provide a reasonable fit to each of the tracers for each sampling campaign. The spatial variance of the models (*c*_0_ + *c*_1_) has a similar pattern to that of the variance of the observations, with a larger spatial variance for the summer sampling campaign and the smallest spatial variance during the winter sampling campaign. In addition, the spatial range of each of the variograms shows a similar pattern, with the effective range (the distance where samples are affected by another) being greatest in winter (particularly for deuterium) when the ranges of the variables were small and the waters were well mixed.

**Figure 6:**
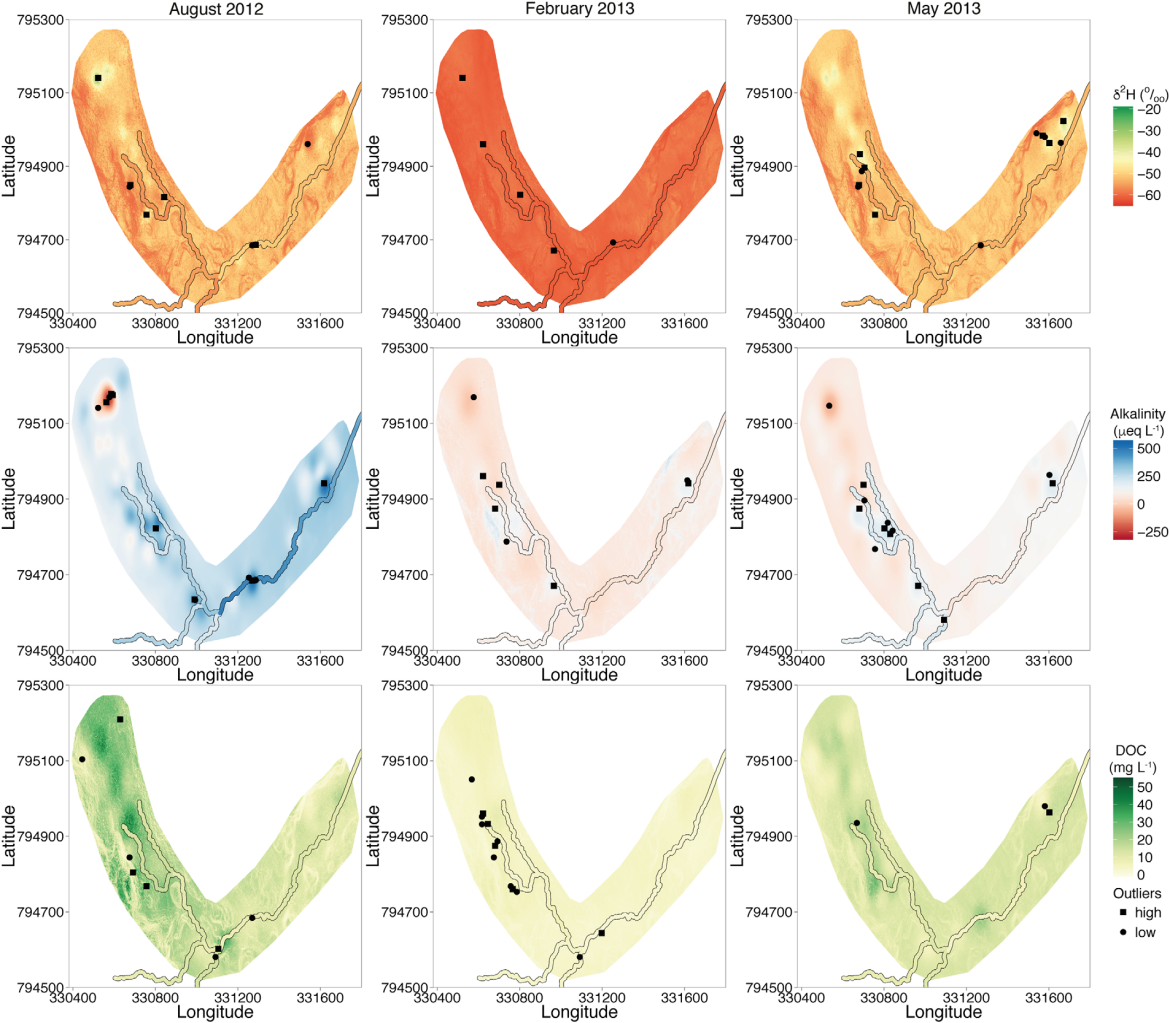
Predicted variables separated into columns to represent the sampling date, and rows to represent the tracer. Location of statistical outliers is also provided on each map which represents localized outliers.

Figure 6 shows the spatial predictions of each tracer in each sampling campaign. In addition, Figure 6 also includes estimates of within stream concentrations based on the sampling points along the stream. During the summer sampling campaign in 2012, there was large spatial variability across the valley bottom for all three tracers. The spatial estimates of δ^2^H closely reflected those of the patterns seen in the point observations (Figure 4). Estimates of GA showed acidic soil waters dominated areas in the WB and the EB in summer while the RZ was predominantly affected by groundwater seeps (i.e., higher GA concentrations) along the stream network. Spatial estimates during February and May showed more uniform and lower concentrations of GA throughout the valley bottom. Similar patterns were seen in May to those observed in August, albeit with more subtle distribution. The spatial patterns of GA and δ^2^H were broadly persistent with regards to locations highlighting areas of groundwater seepage and nonmixing soil water areas. However, the soil water location in the EB was seen in the GA predictions for winter, but it was not so obvious in the February sampling campaign. Concentrations of DOC within the valley bottom indicated that the peaty soils in the WB had high DOC concentrations and lower concentrations in the RZ. The concentrations were uniformly low in February, while in May, there were similar patterns to those of August, with lower concentrations. Looking at the estimates within the stream, it is noticeable that across all tracers, for all sampling periods, there was very little variability compared with that of the potential pool water spatial predictions. The within the stream estimates of all tracers showed that the lower reach of the main stream was generally similar to that of the adjacent surface waters in the riparian zone.

The location and form of the spatial outlier observations are also indicated in Figure 6 with the predictions of each sampling date and variable. In addition, summaries of the outliers are provided in Table 3. The majority of the δ^2^H and GA outliers were positioned in localized areas. For example, there were several outliers of both tracers in an area associated with soil water values in the upper part of the WB. One GA observation in this area was classified as low for each of the sampling campaigns, while a high outlier of δ^2^H was found in this area; both indicated localized soil water in this region. DOC outliers contrast those of the other two tracers as most of its outliers occurred on the borders of the two peat bog areas, often associated with potential groundwater seepages. δ^2^H and GA had the most outliers (13 each) during the winter sampling campaign, whereas DOC had the most outliers during the early summer period (May 2013). The locations and existence of the outliers were not consistent across all tracers and time, which would be expected given the dynamic nature of the mixing of the waters in the valley bottom.

**Table 3:** Mean Values of Classified Low and High Outliers and Nonoutliers for Each Variable With Sample Sizes Also Provided (in Parenthesis)

Variable	Sample Date	Low	Non	High
Deuterium (^o^/_oo_)	Aug 2012	−58.5 (3)	−51.6 (75)	−45.5 (5)
	Feb 2013	−61.8 (1)	−59.7 (87)	−55.0 (4)
	May 2013	−56.6 (6)	−50.9 (81)	−43.0 (7)
Gran alkalinity (µeq L^−1^)	Aug 2012	114.1 (5)	237.8 (71)	495.7 (7)
	Feb 2013	3.4 (3)	64.5 (86)	245.5 (5)
	May 2013	−2.8 (6)	85.7 (80)	445.6 (7)
DOC (mg L^−1^)	Aug 2012	5.2 (4)	18.1 (78)	38.4 (4)
	Feb 2013	3.1 (8)	5.1 (81)	8.9 (5)
	May 2013	7.5 (2)	13.3 (57)	19.1 (1)

### 3.3 Model Evaluation

A summary of the validation of the fixed effects and the random effects is given in Table 4. The accuracy (RMSE) was improved for all models by including the random effects component. In addition, the temporal model with the lowest RMSE for all tracers was for the winter sampling trip. This reduction for the winter models is most likely due to the smaller variability in all tracers during this time period when the saturated area was most extensive and well mixed. The *r* ^2^ value was also improved for each model with the inclusion of the random effects term. The DOC model was best performing (*r* ^2^ = 0.5). The δ^2^H *r* ^2^ values were also acceptable and ranged from 0.3 to 0.44. However, the GA predictions were poorer, with *r* ^2^ values ranging from 0.18 to 0.26. This lack of a good fit for the GA is likely due to more heterogeneity in the composition of groundwater as different sources varied in terms of the weatherability of minerals, which is not reflected by the topographic indices. Validation of the distribution of the predicted random effect based on the theta statistic shows that the use of Dowd's estimator in conjunction with the Matérn function was able to provide reasonable estimates of spatial correlation in the presence of outliers.

**Table 4:** Summary Statistics (RMSE, r^2^, PBIAS, and θ) of Model Validation for the Fixed Effect Model and the Combined Fixed and Random Effect Models

Variable	Sample Date	RMSE (Fixed Effect)	RMSE (Fixed Effect + Random Effect)	*r* ^2^ (Fixed Effect)	*r* ^2^ (Fixed Effect + Random Effect)	PBIAS (Fixed Effect + Random Effect)	$\theta_{mean}$	$\theta_{median}$
Deuterium (^o^/_oo_)	Aug 2012	1.73	1.47	0.22	0.44	0.5	1.38	0.48
	Feb 2013	0.96	0.86	0.24	0.39	−0.4	1.17	0.41
	May 2013	1.62	1.43	0.12	0.33	0.4	1.56	0.38
Gran alkalinity (µeq L^−1^)	Aug 2012	8.26	7.92	0.05	0.19	−0.2	3.52	0.46
	Feb 2013	5.49	4.86	0.07	0.26	0	1.82	0.37
	May 2013	7.52	6.88	0.03	0.18	−0.3	2.09	0.41
DOC (mg L^−1^)	Aug 2012	0.62	0.59	0.27	0.33	−0.2	1.17	0.46
	Feb 2013	0.41	0.35	0.14	0.38	−0.3	1.9	0.54
	May 2013	0.6	0.43	0.04	0.5	0	0.89	0.41

### 3.4 Classification of Water Sources and Mixing in a Riparian Wetland

**Figure 7:**
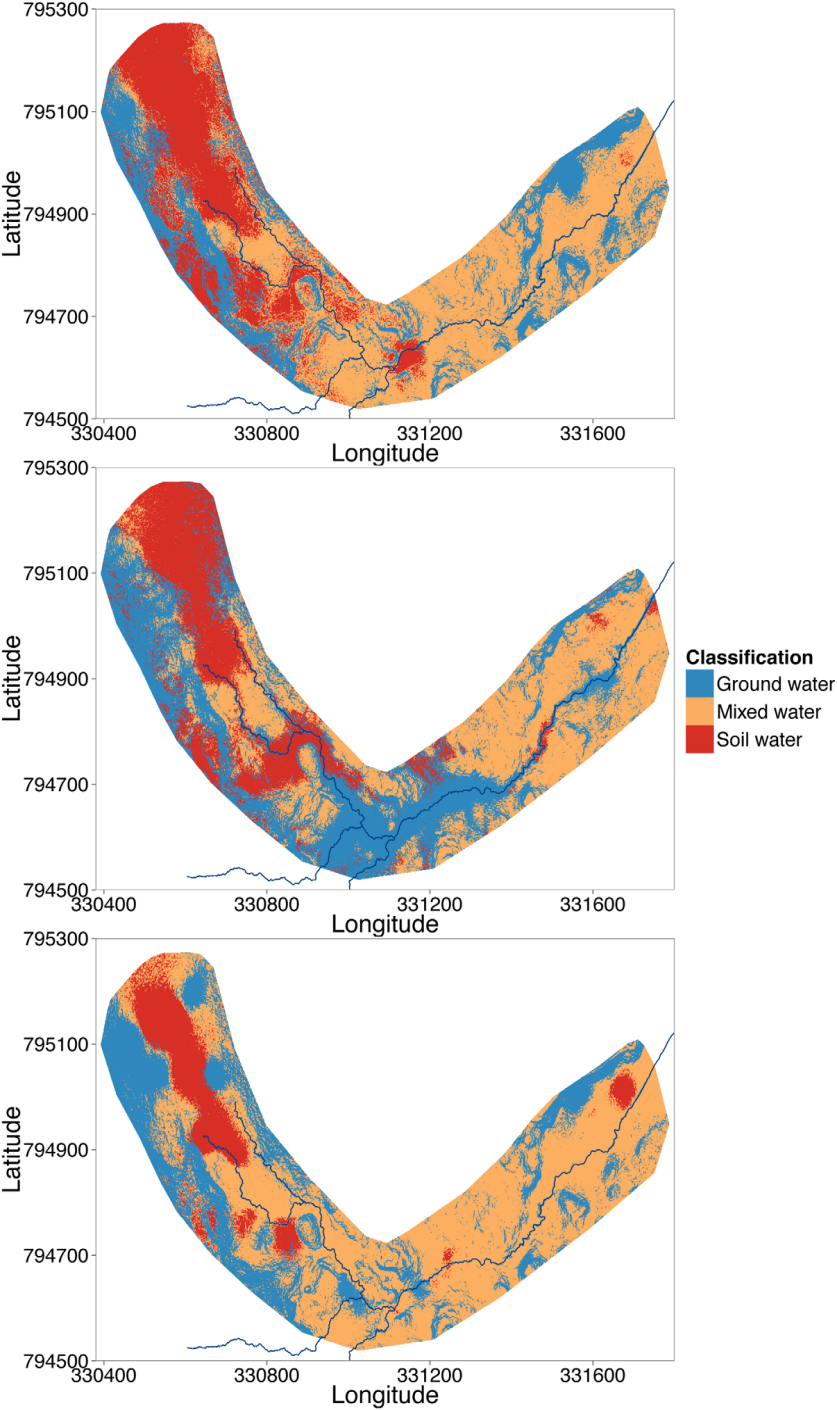
Distribution of the three identified classes of water sources and mixing within the valley bottom for each of the sampling campaigns ((a) August 2012, (b) February 2013, and (c) May 2013).

*k*‐means clustering was undertaken to examine the distribution of the types of water within the riparian area. Figure 7 presents the classified areas within the valley bottom for each of the sampling campaigns. The classification resulted in three main zones; groundwater‐dominated water was characterized by depleted δ^2^H values, high GA concentrations, and low DOC concentrations. This contrasted with soil water sources, which were characterized by enriched δ^2^H values, low GA concentrations, and high DOC concentrations. A third class had intermediate concentrations where integrated water sources reflected mixing of ground and soil water characteristics.

Groundwater seepages on the hillslopes surrounding the WB were persistent across each sampling campaign while the location of soil water within the WB was consistent across all sampling campaigns, but was most evident in late summer (August 2012) and winter (February 2013). Extensive groundwater influence could be observed around the confluences into the RZ during February, which limited the coverage of mixed waters in the RZ. In addition, this expansion of groundwater seepages was reflected by the δ^2^H observed at the outlet of the catchment when the signal was very depleted at the same time (Figure 3). Groundwater seepages were also dominant on the hillslopes north of the EB during each of the sampling campaigns. Mixed waters were predominant in early summer (May 2013) where the vast majority of the valley bottom was classified as mixed waters.

## 4 Discussion

### 4.1 Tracers‐Based Insights on Spatial Heterogeneity of Riparian Water Sources

Many studies have successfully used isotopes or other individual tracers at the catchment outlet to constrain transit time estimates [*Dunn et al*., 2010; *Hrachowitz et al*., 2015; *McGuire et al*., 2007; *Timbe et al*., 2014] and flow path identification [*Kirchner et al*., 2000; *Peters et al*., 2013]. However, it is increasingly recognized that there is a requirement to combine multiple tracers, with their different information content, in a spatially distributed sampling framework to increase the understanding of the role of different water sources on downstream water composition [*Batlle‐Aguilar et al*., 2014; *Fröhlich et al*., 2008; *Zimmer et al*., 2013]. It is apparent from the results of this study that for each tracer, there is marked spatial variability within the extended riparian peatland.

The spatial classifications of the surface waters revealed areas of depleted δ^2^H were generally consistent with areas of high GA and low DOC, indicating deeper minerogenic sources of groundwater that are preferentially recharged during the winter. In contrast, soil water sources tended to be more enriched in δ^2^H as a result of fractionation (and the influence of summer precipitation); higher in DOC and low GA due to provenance in organic soils. These results reinforce the importance of the spatial synoptic sampling and the provide invaluable insight into the mixing that occurs in riparian areas and the processes that integrate to damp variations in stream water composition [*Grabs et al*., 2012; *Zimmer et al*., 2013]. The methods outlined in this study benefitted from previous studies to classify the water types in the catchment. However, it would be possible for these methods to be applied in other catchments where general information on water sources is available.

### 4.2 Integration of Geostatistical Tracer Analysis and LiDAR to Classify Riparian Source Areas and Mixing Zones

Heterogeneity within source areas has been shown to have a large influence on downstream chemistry [*Ågren et al*., 2014; *Nadeau and Rains*, 2007; *Zimmer et al*., 2013]. Geospatial tools are increasingly being used to examine spatial trends within stream networks based on the distance and connectivity of the stream networks [*Hoef et al*., 2006] and in a terrestrial context based on Euclidean space [*Bivand et al*., 2008]. In this study, we have utilized regression kriging [*Odeh et al*., 1995] to estimate the spatial trends of each tracer under different hydroclimatic conditions within these source areas, where the spatial estimates represent the expected value of each tracer, if surface water was present.

Many of the fitted models had relatively low *r* ^2^ values, with elevation being the dominant topographic index being included in the models. The results of the backward elimination suggest the SWI (similar to the topographic wetness index [*Böhner and Selige*, 2006], used in TOPMODEL [*Beven and Freer*, 2001]) did not have a strong influence on the distribution of the tracers and was only included in one of the nine models. Thus, many of the topographic indices were not related to any of the tracers and therefore, did not help to explain the heterogeneity of the surface waters. These results are supported by previous studies which have shown that topology alone is not a good predictor of hydrological functioning in these glacial landscapes with wide valley bottoms and ground water seeps [*Ali et al*., 2014; *Hinton et al*., 1993; *Inamdar and Mitchell*, 2006; *Tetzlaff et al*., 2008].

Using the methods outlined in *Lark* [2002], it was possible to identify outlying observations. The results of this analysis revealed that the presence of δ^2^H and GA outliers were present in areas of soil water with one sampling site in particular showing low GA on each visit. In contrast, the results of the DOC outliers occurred mainly near the bordering areas of the peat bogs, which is likely to be due to groundwater influxes from known groundwater seepages and the heterogeneity of the extended riparian zones. However, it is important to note that the majority of the detected outliers in the catchment were not consistent through time and therefore influenced by other factors including climatic conditions and antecedent conditions, reinforcing the importance of hydroclimatic changes on the source waters [*Grabs et al*., 2012; *Orlowski et al*., 2015].

### 4.3 Hydroclimatic Influences and Temporal Dynamics on Spatial Patterns of Water Sources and the Hydrological Functioning

Temporal dynamics have been shown to have a large influence on saturation extent within the riparian zone of the study catchment [*Birkel et al*., 2010; *Inamdar and Mitchell*, 2006] and have a large influence on the chemistry of streams [*Ali et al*., 2014; *Hooper and Aulenbach*, 1998; *McGlynn and Seibert*, 2003]. Our results of the synoptic survey support these previous findings, and increase the understanding of how the changes in hydroclimatic and subsequently saturation extent control the spatial variability of the tracers and linked source waters within the riparian zone. The high‐resolution surveys revealed that heterogeneity of the source waters was present at all times, but more pronounced during warmer and dryer periods, when sources of water were more disconnected. The extent of the classified areas of soil, mixed, and groundwater is reflected in the temporal dynamics observed at the catchment outlet, with greater temporal variability during warmer months, contrasted with relatively little variability during cooler months. This relationship between the spatial variability and temporal dynamics observed at the catchment outlet reinforces the importance of considering catchment heterogeneity when trying to understand hydrological functioning of systems [*Grabs et al*., 2012; *Orlowski et al*., 2015]. This tight relationship reaffirms the difficulty in understanding how the variability of source waters affects downstream chemistry [*Batlle‐Aguilar et al*., 2014].

## 5 Conclusion

In this study, we applied geospatial methods in conjunction with multiple environmental tracers to examine the dynamics and mixing of source water within an extended riparian zone. Our results have revealed the highly heterogeneous and dynamic nature of the source waters in extended riparian zones, which is not evident at the catchment outlet and would be missed if sampling would only take place at the outlet. In addition, there was no consistent relationship between commonly derived topographic indices and the spatial variability of the tracers which supports previous studies in such glaciated landscapes, where other controls than topography (such as soil distribution and landscape evolution) are dominating the hydrological response. This work highlights the complexity of understanding this relationship and the value of integrated approaches given the dynamic nature of the source waters through time and space.
